# Human adipose mesenchymal stem cells modulate inflammation and angiogenesis through exosomes

**DOI:** 10.1038/s41598-022-06824-1

**Published:** 2022-02-17

**Authors:** June Seok Heo, Sinyoung Kim

**Affiliations:** 1 Cell Therapy Center, Severance Hospital, Seoul, 03722 Republic of Korea; 2 Department of Laboratory Medicine, Yonsei University College of Medicine, Seoul, 03722 Republic of Korea

**Keywords:** Cell biology, Stem cells

## Abstract

Stem cell-derived exosomes are efficient and safe therapeutic tools for transferring endogenous biological cargo or functional biomolecules for regenerative medicine. The regulation of inflammation and angiogenesis plays a pivotal role in wound healing and tissue regeneration. The purpose of this study was to investigate the anti-inflammatory and pro-angiogenic roles of human adipose mesenchymal stem cell-derived exosomes, focusing on the underlying mechanisms. Exosomes inhibited LPS-induced inflammation by activating *ROCK1* and *PTEN* expression. Moreover, microRNAs (miR-132 and miR-146a) released from exosomes upregulated the expression of pro-angiogenic genes and promoted proliferation activity and tube formation in human umbilical vein endothelial cells. Exosomal effects were verified using ROCK1/PTEN inhibitors for anti-inflammation and miR-132/miR-146a inhibitors for pro-angiogenesis. Our findings suggest that exosomes exert anti-inflammatory effects by targeting the ROCK1/PTEN pathway and exhibit pro-angiogenic effects via delivery of miR-132 and miR-146a. Taken together, these results suggest that exosomes may be promising therapeutic candidates for curing diseases involved in inflammation and angiogenesis.

## Introduction

Accumulating evidence indicates that stem cell-based therapies are effective in the treatment of intractable disorders, including degenerative, immune, and genetic disorders, as well as COVID-19^1,2^. Stem cells act in their microenvironment and surrounding cells by differentiating into target cells or releasing cytokines, growth factors, and beneficial biomolecules^3^. Of these, the therapeutic effects of exosomes of paracrine factors have recently attracted significant interest in regenerative medicine because exosomes are much easier to handle than cells^4^. Recently, the major mechanism of stem cell therapy has been attributed to exosome-based paracrine effects rather than cell differentiation or replacement through migration and homing^5,6^. Exosomes have a variety of merits, including no tumorigenicity, low immune reactions, and fewer ethical issues^7^. More importantly, exosomes are safe agents for clinical trials as they are cell-based cell-free agents and do not induce side effects^8^. Exosomes are nano-sized (40–120 nm) membrane-bound extracellular vesicles that originate from the endocytic pathway of eukaryotic cells. Exosomes play an important role in intercellular communication by transferring biomolecules such as proteins, nucleic acids, mRNAs, microRNAs (miRNAs), and other non-coding RNAs^9^. Recently, mesenchymal stem cell (MSC)-derived exosomes have been extensively studied and shown to contribute to clinical application^10^. A variety of studies have shown that exosomes are effective in attenuating injury, improving neurological recovery, and facilitating wound regeneration^11–13^.

Various disorders, such as wounds and inflammatory diseases, impact repair processes, including inflammation, proliferation, and angiogenesis^14,15^. These processes involve complex and specific interactions between diverse cell types, cytokines, mediators, and cell signaling^16^. Exosomes have been shown to inhibit excessive immune responses through various mechanisms, including the regulation of immune cells such as macrophages and T cells, indicating that exosomes have potential as therapeutic agents for inflammatory modulation^17,18^. Additionally, MSC-based cell therapies have been increasingly used to improve angiogenesis^19^. Recent studies have indicated that MSCs promote angiogenesis through exosomes^20^. For example, the transplantation of exosomes enhanced bone repair by improving angiogenesis in a rat model^21^. Our previous studies showed that human adipose tissue (AdMSC)-derived exosomes inhibit inflammation by promoting M2 macrophages and delivering miRNAs^22,23^. AdMSC-exosomes also enhanced wound healing by attenuating pro-inflammatory responses^24^. Understanding how AdMSC-exosomes communicate with target cells in their surrounding microenvironment is a major issue for diseases involving inflammation and angiogenesis. In this study, we investigated the mechanisms underlying the anti-inflammatory and pro-angiogenic effects of AdMSC-exosomes using THP-1 cells treated with lipopolysaccharide (LPS) and human umbilical vein endothelial cells (HUVECs). We performed experiments to examine the modulation of pro-inflammatory and anti-inflammatory factors by AdMSC-exosomes. ROCK1 (Rho associated coiled-coil containing protein kinase 1), which mediates immune cells, functions as a suppressor of inflammation by regulating phosphatase and tensin homolog (PTEN)^25^. PTEN also suppresses inflammation via the Wnt/β-catenin pathway Subsequently, the expression of *ROCK1* and *PTEN* by AdMSC-exosomes in inflammation-stimulated environments was assessed. We also identified specific exosomal miRNAs that improve HUVEC function, including proliferation activity and tube formation.

## Results

### AdMSCs and AdMSC-derived exosomes exhibit typical characteristics

**Figure 1:**
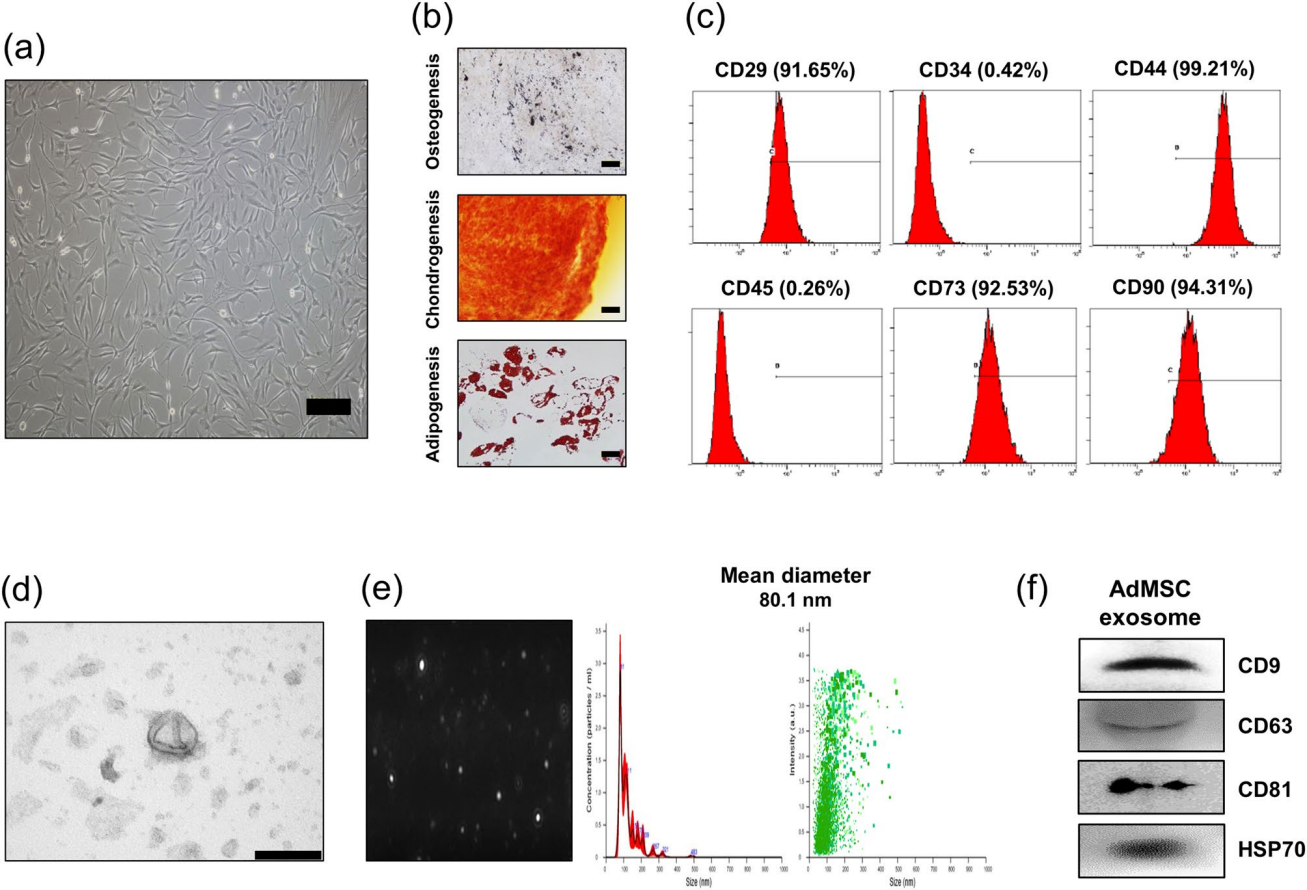
Characterization of AdMSCs and AdMSCs-derived exosomes. (**a**) The morphology of cultured cells was determined by microscopy. The cells displayed the typical spindle-shaped morphology of MSCs (× 100 magnification, scale bar = 200 μm). (**b**) To confirm whether cultured cells are MSCs, cells were induced into osteocytes, chondrocytes, and adipocytes for 3 weeks. Osteogenic (× 200 magnification, scale bar = 100 μm), chondrogenic (× 200 magnification, scale bar = 100 μm), and adipogenic (× 400 magnification, scale bar = 50 μm) differentiation were verified by von Kossa, Safranin-O, and Oil red O staining, respectively. (**c**) Analysis of surface markers on MSCs revealed that cells were positive for CD29, CD44, CD73, and CD90 (MSCs markers), while negative for CD34 and CD45 (hematopoietic markers). (**d**) The image of AdMSCs-derived exosomes was obtained via transmission electron microscopy (scale bar = 200 nm). (**e**) Size distribution of exosomes was determined by Nanosight. The mean diameter was 80.1 nm. (**f**) CD9, CD63, CD81 and HSP70 of exosomal markers were detected in AdMSCs-derived exosomes by Western blot assay.

Cultured cells had spindle-shaped and fibroblast-like MSC morphology (Fig. 1a). To evaluate the differentiation capacity of cultured MSCs, the cells were induced into osteocytes, chondrocytes, and adipocytes in a specific environment. Induced cells were strongly differentiated into osteocytes, chondrocytes, and adipocytes as stained with von Kossa, safranin O, and oil red O, respectively (Fig. 1b). To further confirm whether cultured cells were MSCs, a flow cytometry assay was used for the analysis of surface proteins. Data showed that the cells were positive for well-known markers of MSCs: CD29 (integrin β1), CD44, CD73 (ecto-5′-nucleotidase), CD90 (thy-1), and negative for markers of hematopoietic cells: CD34, and CD45 (Fig. 1c). These results demonstrate that cultured cells are MSCs.

To characterize AdMSCs-derived exosomes, we first observed the morphology of the isolated exosomes. TEM image showed that the exosomes had round membrane-bound vesicles (Fig. 1d). The mean diameters of exosomes were confirmed as 80.1 nm through nanoparticle tracking analysis (Fig. 1e). Furthermore, CD9, CD63, CD81 and HSP70 of common exosomal markers were detected in isolated exosomes by western blotting (Fig. 1f). These results reveal that AdMSC-derived exosomes have typical exosome features.

### AdMSC-derived exosomes inhibited the inflammatory response by modulating key factors in pro-inflammation and anti-inflammation

**Figure 2:**
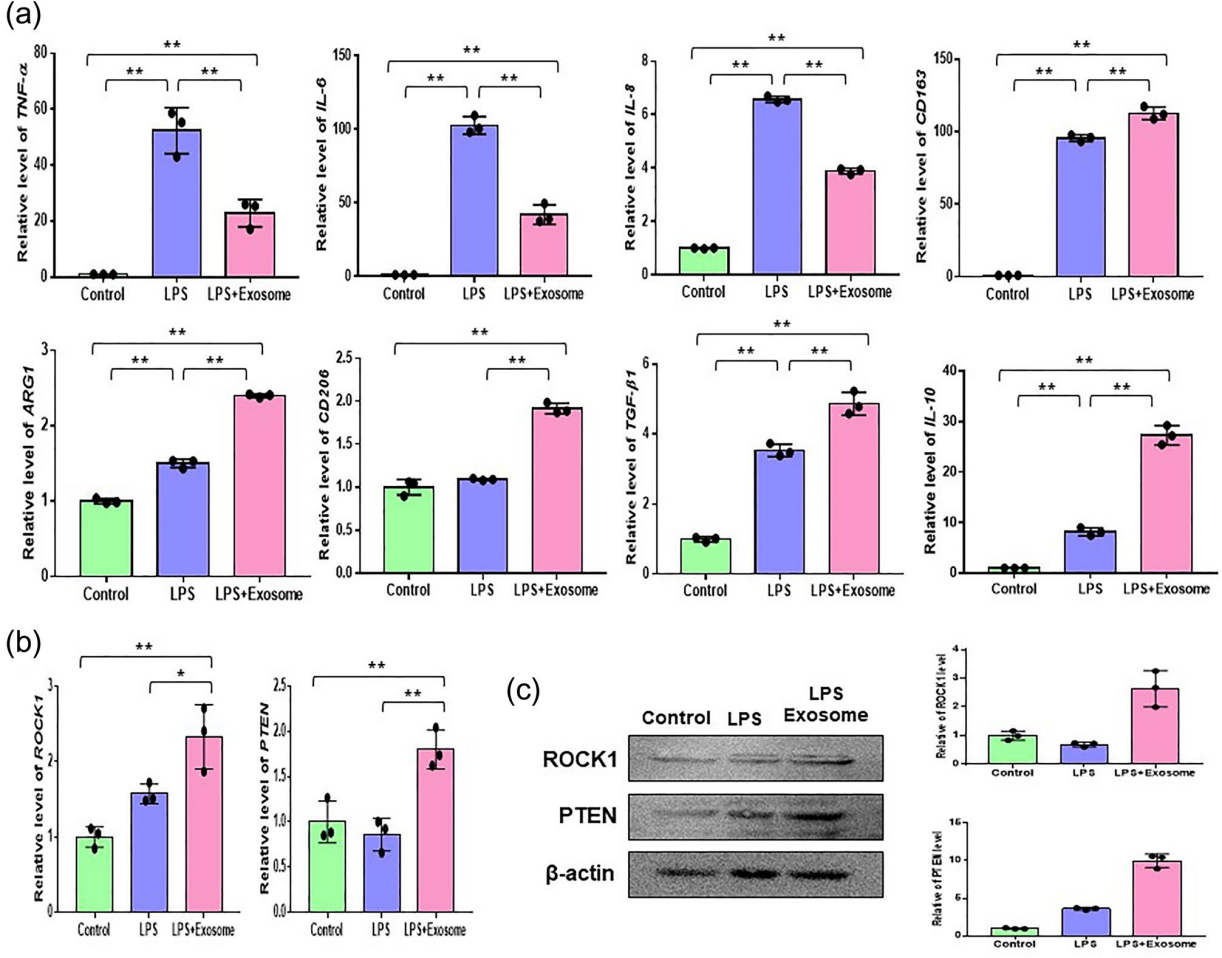
Effect of AdMSCs-derived exosomes on the inflammatory response. (**a**) THP-1 cells were treated with 100 ng/mL LPS and LPS + 5 μg/mL exosomes for 24 h. Untreated cells were used as controls. Real time-qPCR was performed to investigate the changes of relative gene expressions on inflammatory- (TNF-α, IL-6, IL-8) and anti-inflammatory mRNAs (CD163, Arg1, CD206, TGF-β1, IL-10). (**b**) The expression of ROCK1 and PTEN was analyzed in THP-1 cells after treatment with LPS and LPS + exosomes for 24 h by real time-qPCR. GAPDH was utilized as an internal control. (**c**) The protein levels of ROCK1 and PTEN were confirmed by Western blotting. β-actin was used as an internal control. The relative values of ROCK1/β-actin and PTEN/β-actin were determined by Image J program. The data are expressed as the mean ± SD of three independent experiments. *Significant difference from untreated control (*p* < 0.05). **Significant difference from untreated control (*p* < 0.01).

To assess the effect of AdMSC-derived exosomes on the inflammatory response, THP-1 cells were treated with 100 ng/mL lipopolysaccharide (LPS) and LPS + 5 μg/mL exosomes. Next, we analyzed the pro-inflammatory (*TNF-α*, *IL-6*, *IL-8*)- and anti-inflammatory (*CD163*, *ARG1*, *CD206*, transforming growth factor *(TGF)-β1*, *IL-10*)-mRNAs by real-time qPCR after incubation for 24 h. We confirmed that the expression of *TNF-α*, *IL-6*, and *IL-8* in the LPS + exosome treated group was remarkably reduced compared to that in the LPS treated group (Fig. 2a). Moreover, we observed that the expression levels of *CD163*, *ARG1*, *CD206*, *TGF-β1*, and *IL-10* in the LPS + exosome treated group were greatly increased compared to those in the control group and LPS treated group (Fig. 2a). These results indicated that AdMSC-derived exosomes could induce anti-inflammatory environments via anti-inflammatory molecules secreted from M2 polarization, showing their potential in inflammation-related diseases.

### AdMSCs-derived exosomes improve anti-inflammatory effect by activating the ROCK1 and PTEN pathways

To explore the mechanisms underlying the anti-inflammatory effects of AdMSC-derived exosomes, we analyzed the expression levels of *ROCK1* and *PTEN*, which play significant roles as suppressors of inflammation. The mRNA levels of *ROCK1* and *PTEN* in the LPS + exosome treated group showed the most significant increase in comparison with those in the control group and LPS treated group (Fig. 2b), which verified the enhancement of ROCK1 and PTEN in the LPS + exosome treated group at the protein level (Fig. 2c). These data showed that exosomes improved the anti-inflammatory effect by reducing pro-inflammatory factors by upregulating *ROCK1* and *PTEN*.

**Figure 3:**
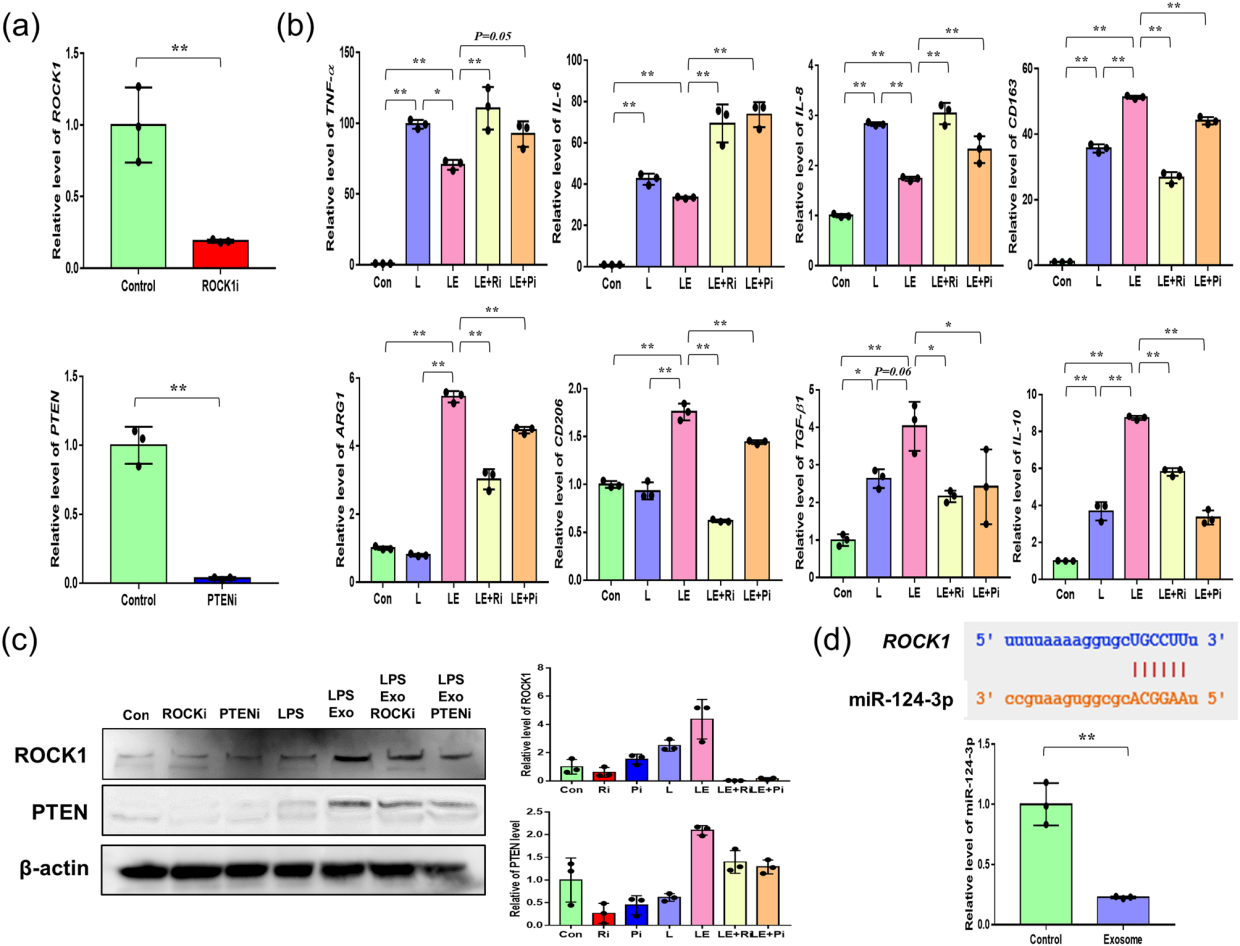
Anti-inflammatory effects of AdMSCs-derived exosomes via ROCK1/PTEN pathway. (**a**) The expression levels of ROCK1 and PTEN were quantified by real time-qPCR after treatment with ROCK1 and PTEN inhibitor. (**b**) THP-1 cells were treated with 100 ng/mL LPS (L), LPS + 5 μg/mL exosomes (LE), LPS + exosomes + 10 nM ROCK1 inhibitor (LE + Ri), and LPS + exosomes + 1 nM PTEN inhibitor (LE + Pi). (**c**) Protein levels of ROCK1 and PTEN were analyzed by western blot. β-actin was used as controls. Relative protein levels were confirmed by quantification. (**d**) A schematic diagram of miR-124-3p binding sites in the 3’ UTR of ROCK1 mRNA. After treatment with exosomes, the relative level of miR-124-3p was determined in THP-1 cells by real time-qPCR. The data are expressed as the mean ± SD of three independent experiments. *Significant difference from untreated control (*p* < 0.05). **Significant difference from untreated control (*p* < 0.01).

To further confirm the effect of the ROCK1 and PTEN pathways on the regulation of inflammation, we applied the ROCK1 inhibitor Y-27632 and PTEN inhibitor SF1670. After inhibition, we confirmed that the expression of *ROCK1* and *PTEN* was significantly decreased by the inhibitors (Fig. 3a). The LPS + exosome treated group demonstrated significantly higher relative expression levels of anti-inflammatory genes (*CD163*, *ARG1*, *CD206*, *TGF-β1*, *IL-10*) and significantly reduced expression levels of pro-inflammatory genes (*TNF-α*, *IL-6*, *IL-8*) compared with the control group and LPS treated group (Fig. 3b). Notably, the above effects of exosomes were significantly inhibited by ROCK1 and PTEN inhibitors, showing that ROCK1 and PTEN inhibitors weakened the anti-inflammatory effect mediated by exosomes (Fig. 3b). Subsequently, the underlying mechanisms of exosomes via ROCK1 and PTEN were measured at the protein level and showed evidence that ROCK1/PTEN act as key modulators through exosomes (Fig. 3c). Based on the previous results, miR-124-3p inhibited the expression of *ROCK1* as predicted by the binding sites of miR-124-3p and *ROCK1* using the Starbase database website (http://starbase.sysu.edu.cn/) (Fig. 3d)^26^. To support the mechanisms of ROCK1 exosome formation, we detected miR-124-3p expression in the exosome treated group. The results showed that miR-124-3p expression was downregulated in the exosome treated group compared to the control group (Fig. 3d). These results indicate that low expression of miR-124-3p increases *ROCK1* expression in exosomes.

### AdMSCs-derived exosomes promote endothelial cell proliferation and angiogenesis

**Figure 4:**
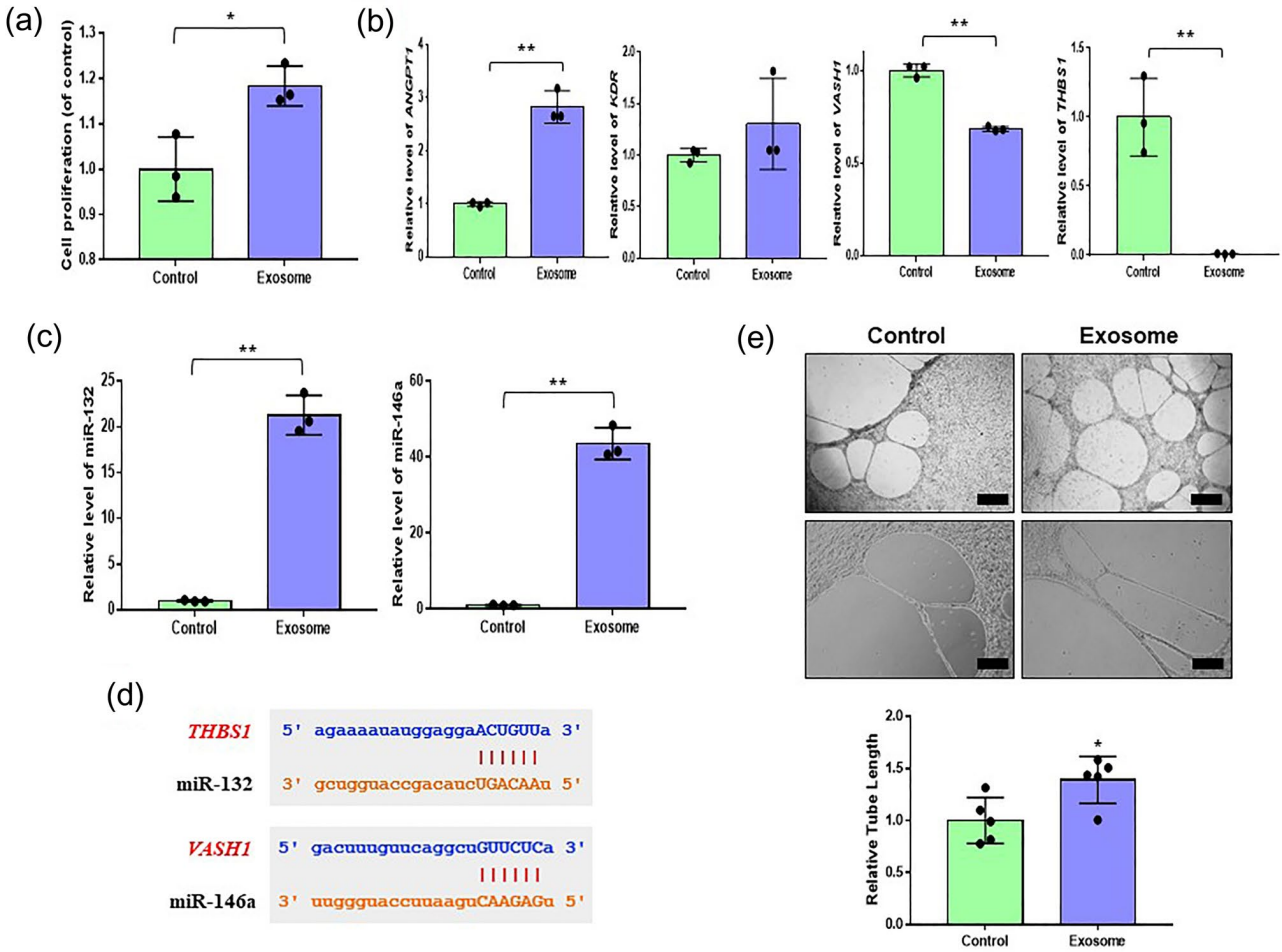
Effect of AdMSCs-derived exosome on angiogenesis. (**a**) Growth rates of HUVEC were analyzed after treatment with exosomes using a proliferation assay kit. (**b**) After HUVECs were treated with exosomes, the mRNA levels of proangiogenic angiopoietin1(*ANGPT1*), flk1(*KDR*), and antiangiogenic vasohibin-1(*VASH1*), thrombospondin-1(*THBS1*) were analyzed by real time-qPCR. (**c**) After incubation with exosomes in HUVECs, relative levels of miR-132 and miR-146a were evaluated by real time-qPCR. (**d**) Schematic diagrams show miR-132 binding sites in the 3’ UTR of THBS1 (Thrombosopndin-1) and miR-146a binding sites in the 3’ UTR of VASH1 (Vasohibin-1) mRNA. (**e**) In vitro tube formation of HUVECs was assayed onto matrigel. Enhanced tube formation was observed in exosomes-treated HUVECs (× 40 magnification, scale bar = 500 μm) (× 100 magnification, scale bar = 200 μm). The data are expressed as the mean ± SD of three independent experiments. *Significant difference from untreated control (*p* < 0.05). **Significant difference from untreated control (*p* < 0.01).

To investigate the effect of exosomes on proliferation activity, HUVECs were treated with 5 μg/mL exosomes for 24 h. Exosomes significantly increased the proliferation of HUVECs (Fig. 4a). Next, we examined the expression levels of pro-angiogenic and anti-angiogenic genes to determine whether exosomes can induce angiogenesis. We found that the expression levels of angiopoietin1(*ANGPT1*) and flk1(*KDR*) (the pro-angiogenic genes) were markedly enhanced, while those of vasohibin-1(*VASH1*) and thrombospondin-1(*THBS1*) (anti-angiogenic genes) were significantly reduced in exosome-treated HUVECs (Fig. 4b). MiR-132 and miR-146a activate angiogenesis^27,28^. To verify the activities of miR-132 and miR-146a by exosomes, we analyzed the expression levels of miR-132 and miR-146a in exosome-treated HUVECs. As expected, the expression levels of miR-132 and miR-146a were remarkably increased in the exosome-treated group (Fig. 4c). Interestingly, we found that miR-132 binds the anti-angiogenic gene, thrombospondin-1(*THBS1*), and miR-146a binds the anti-angiogenic gene, vasohibin-1(*VASH1*), using the Starbase database website (Fig. 4d). These data indicate that interactions between reduced anti-angiogenic genes (vasohibin-1(*VASH1*), thrombosopondin-1(*THBS1*)) and enhanced miRNAs (miR-132, miR-146a) act as key factors in angiogenesis. Based on the above results, we further analyzed the effects of exosomes on angiogenesis by performing a tube formation assay. The results showed that tube formation by exosome-treated HUVECs was significantly enhanced, as shown in Fig. 4e. Taken together, our results show that AdMSC-derived exosomes promote angiogenesis of HUVECs by upregulating pro-angiogenic genes and downregulating anti-angiogenic genes.

### AdMSCs-derived exosomes improve endothelial cell angiogenesis via miR-132 and miR-146a

**Figure 5:**
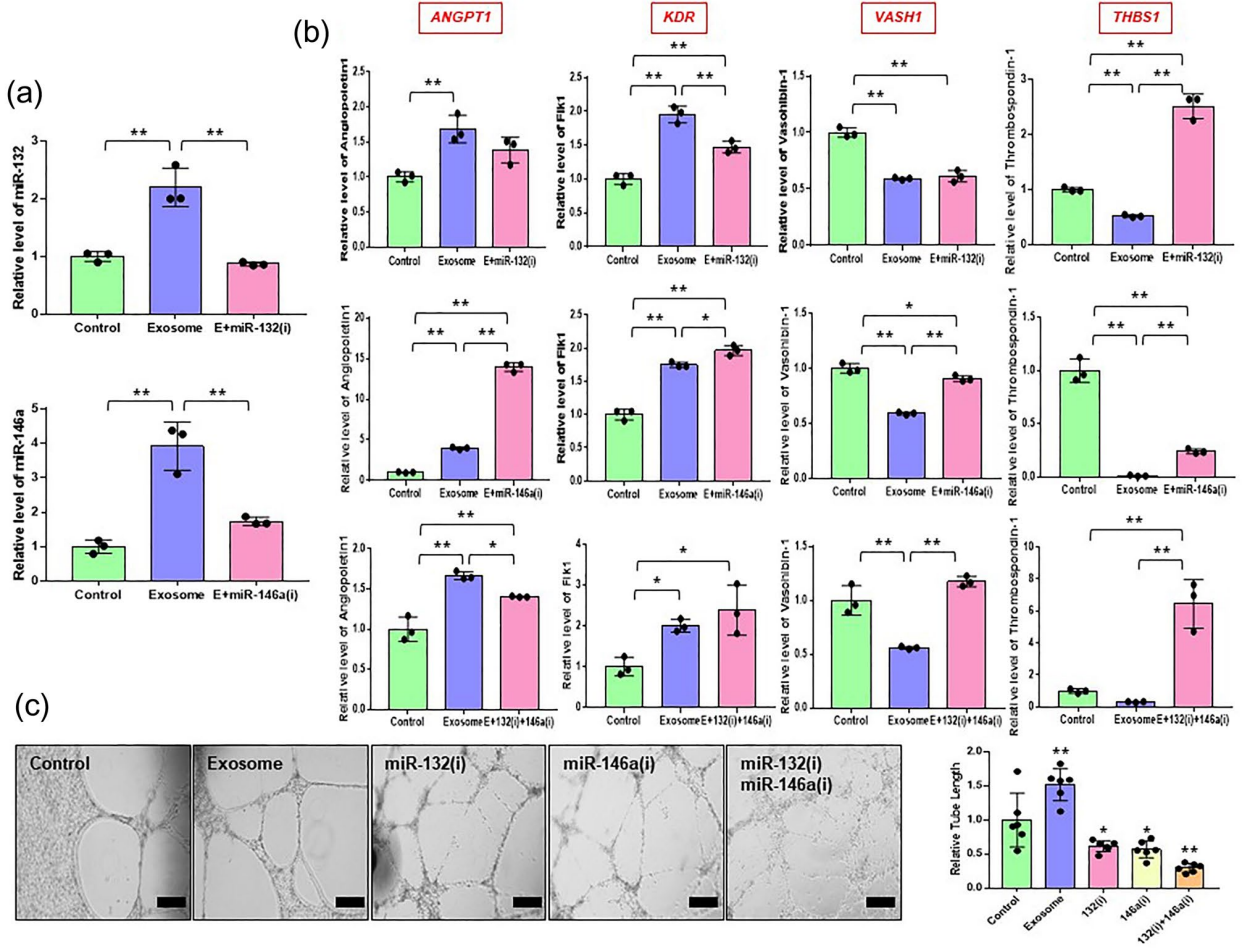
miR-146a and miR132 involve angiogenesis of HUVECs. (**a**) The expression of miR-146a and miR-132 in HUVECs transfected with the miR-146a (miR-146a(i)) or miR-132 inhibitor (miR-132(i)), as determined by real time-qPCR. (E; exosome) (**b**) The expression levels of vasohibin-1(*VASH1*), thrombospondin-1(*THBS1*), angiopoietin1(*ANGPT1*), and flk1(*KDR*) in HUVECs transfected with the miR-146a or/and miR-132 inhibitor, as evaluated by real time-qPCR. (**c**) Tube formation in HUVECs transfected with the miR-146a or/and miR-132 inhibitor was determined using matrigel assay (× 100 magnification, scale bar = 200 μm). The data are expressed as the mean ± SD of three independent experiments. *Significant difference from untreated control (*p* < 0.05). **Significant difference from untreated control (*p* < 0.01).

To study the role of miR-132 and miR-146a in angiogenesis, we transfected HUVECs with miR-132 and miR-146a inhibitors. As shown in Fig. 5a, the relative expression of miR-132 and miR-146a was markedly enhanced by exosomes, whereas the expression was significantly decreased by miR-132 and miR-146a inhibitors. Next, the expression levels of pro-angiogenic and anti-angiogenic genes were measured using real-time qPCR. Overall, the results showed that angiopoietin1(*ANGPT1*) and flk1(*KDR*) expression of pro-angiogenic genes were increased in the exosomes and inhibitor groups regardless of inhibitors (Fig. 5b). Notably, we found that miR-132 inhibitor transfection increased thrombospondin-1(*THBS1*) expression levels (Fig. 5b). In addition, miR-146a inhibitor transfection enhanced the vasohibin-1(*VASH1*) expression level decreased by exosome treatment (Fig. 5b). To validate the role of miR-132 and miR-146a, we co-transfected HUVECs with miR-132 and miR-146a. We found that the expression levels of angiopoietin1(*ANGPT1*) and flk1(*KDR*) were increased in both the exosome and inhibitor groups regardless of inhibitor co-transfection (Fig. 5b). Interestingly, the relative levels of vasohibin-1(*VASH1*) and thrombospondin-1(*THBS1*), known as anti-angiogenic genes, were reduced by exosomes, while inhibitors reversed the effect of exosomes (Fig. 5b). These results revealed that enhancing miR-132 and miR-146a may promote angiogenesis in HUVECs.

We further analyzed the roles of miR-132 and miR-146a on angiogenesis in vitro using a tube formation assay. As confirmed in Fig. 4e, tube formation of HUVECs was improved in the exosomes group, while the weakness of tube formation was observed in miR-132 and miR-146a inhibitor groups, although tube-like structures were formed (Fig. 5c). The formation of tube-like structures was even destroyed in the miR-132 and miR-146a inhibitor co-transfected group (Fig. 5c). Taken together, our data indicate that miR-132 and miR-146a are critical factors in the pro-angiogenic activity of HUVECs.

## Discussion

In recent years, the application of extracellular vesicles, including exosomes derived from MSCs, has gained significant interest in stem cell research because their use shows effects similar to those of originated cells^29^. Importantly, the use of these vesicles can be free from many potential risks, such as immune rejection and tumor formation^30^. In this study, we evaluated the roles of AdMSC-derived exosomes and their potential to improve inflammation and angiogenesis. In particular, the underlying mechanisms were investigated, including signaling pathways and candidate miRNAs between exosomes and target cells. Liu et al. previously reported that the anti-inflammatory effects of MSC-derived exosomes were mediated by upregulating PTEN^31^. ROCK1, which functions as an inhibitor of inflammation, plays a critical role in the regulation of PTEN activation^32^. Thus, we aimed to elucidate the crosstalk between ROCK1 and PTEN by exosomes in an inflammation-induced environment. Our results revealed that AdMSC-derived exosomes suppressed LPS-induced inflammation via the ROCK1 and PTEN pathways. Moreover, we validated the exosomal effects on inflammatory responses using ROCK1 or PTEN inhibitors.

Inflammation is closely associated with tissue repair and regeneration, especially wound healing^33^. Tissue regeneration is affected by fibroblasts, immune cells, MSCs, and endothelial cells, which upregulate pro-angiogenic factors to induce angiogenesis and inhibit inflammation^34^. Therefore, we investigated whether AdMSC-derived exosomes enhance the proliferation and tube formation of HUVECs in vitro, which are significant for vascularization. Our results showed that AdMSC-derived exosomes markedly enhanced proliferation and tube formation in terms of angiogenesis in HUVECs. These results support that AdMSC-derived exosomes are an attractive therapeutic tool for promoting angiogenesis and wound repair. We next examined the mechanism responsible for the pro-angiogenic ability of AdMSC-derived exosomes in exosome-treated HUVECs. Generally, miRNAs function as negative regulators of gene expression. Specific miRNAs differentially regulate pro- or anti-angiogenic activities depending on cell or tissue types^35^. For example, miR-132 and miR-146a are known to be angiogenic enhancers^28,36^: miR-132 induces angiogenesis and miR-146a promotes angiogenesis by enhancing the functionality of endothelial progenitor cells in mice^27,37^. However, the functions of miR-132 and miR-146a in angiogenesis-related genes in HUVECs remain unclear. In this study, miR-132 and miR-146a were highly expressed in exosome-treated HUVECs. Subsequently, we confirmed that miR-132 and miR-146a bind thrombospondin-1(*THBS1*) and vasohibin-1(*VASH1*) of anti-angiogenic genes, respectively. To explore the effect of miR-132 and miR-146a on angiogenesis in more detail, we investigated the expression of angiogenesis-related genes using miR-132 and miR-146a inhibitors. We found that the expression of anti-angiogenic genes was remarkably reduced with exosome incubation, while that of the genes was reversed by miR-132 and miR-146a inhibitors. The inhibition of miR-132 and miR-146a leads to poor angiogenic ability of HUVECs. Based on our findings, we suggest that miR-132 and miR-146a are the principal players in the effects of AdMSC-derived exosomes on angiogenic activity, and exert their effect by inhibiting anti-angiogenic genes.

In this study, we re-identified that the expression of anti-inflammatory factors related to M2 macrophages was significantly enhanced, consistent with previous studies showing that AdMSC-derived exosomes inhibit inflammation by upregulating M2 macrophages^24^. Here, we focused on the signaling pathways activated by exosomes in the target cells. Our results showed that AdMSC-derived exosomes enhanced the expression of *ROCK1* and *PTEN* for anti-inflammation. However, other signaling regulators may also be associated with the ROCK1/PTEN process by exosomes, and it is necessary to examine how other inflammatory signaling pathways regulate the immune response in depth. Many studies have shown that multiple miRNAs are involved in angiogenesis^38^. In this study, we predicted that specific miRNAs might modulate the angiogenesis of HUVECs by AdMSC-derived exosomes. Indeed, we found that inhibition of miR-132 and miR-146a in HUVECs diminished their pro-angiogenic activity. To our knowledge, this is the first study to demonstrate the regulation of anti-angiogenic genes by miR-132 and miR-146a transferred from AdMSC-derived exosomes. Although our study demonstrates the role of miR-132 and miR-146a in angiogenesis by exosomes, further studies are required to determine the overall relationship between miRNAs and the wider secretome.

**Figure 6:**
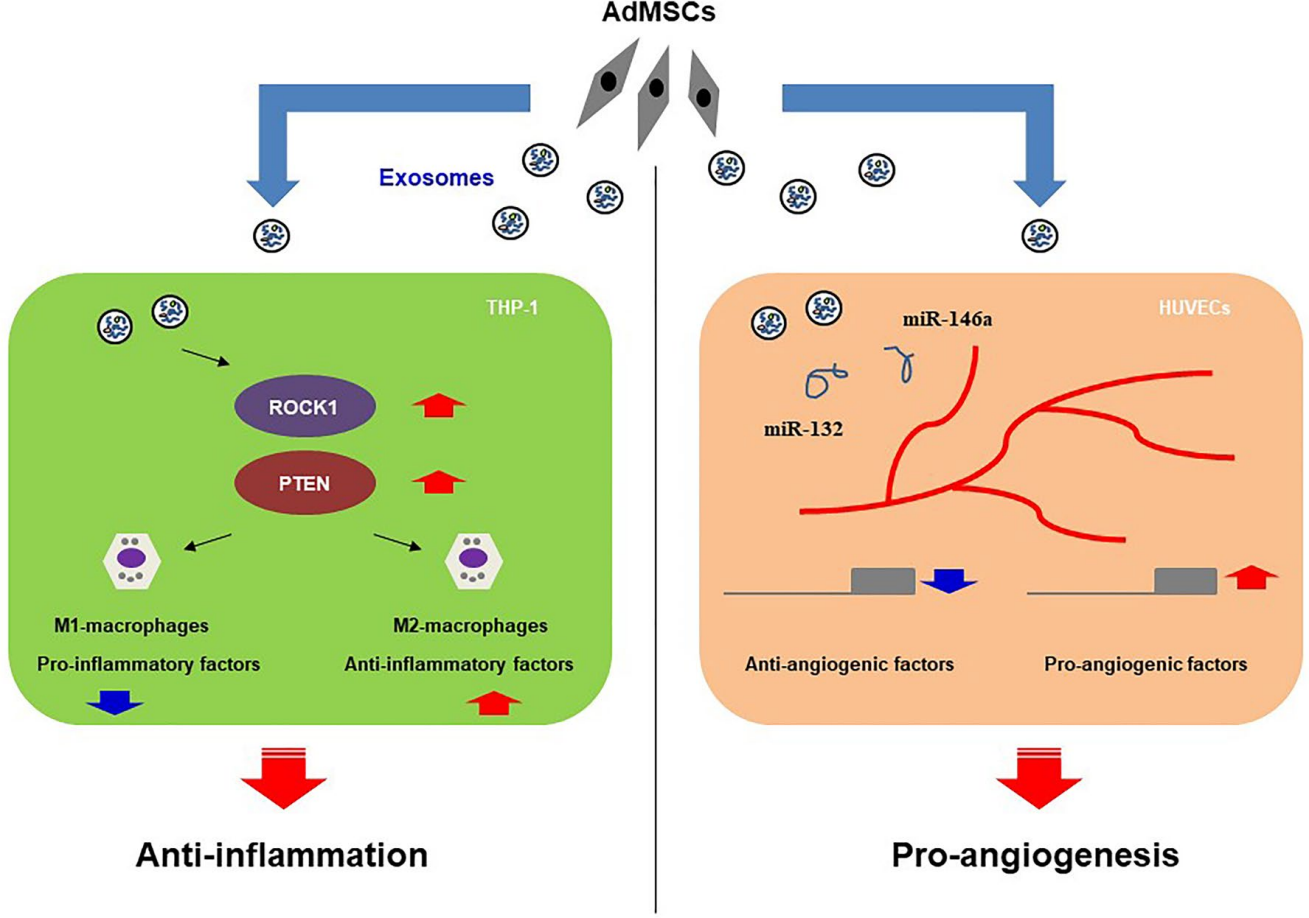
A schematic diagram depicts the positive effects of AdMSCs-derived exosomes on inflammatory responses of THP-1 cells and angiogenesis of HUVECs.

Based on stem cell therapy using exosomes, we demonstrated that high expression of *ROCK1*/*PTEN* in THP-1 cells is responsible for modulating inflammation, and high levels of miR-132/miR-146a are responsible for regulating angiogenesis in HUVECs by downregulating anti-angiogenic genes. In summary, we described a new finding of exosomal effects as immunosuppressant and angiogenic inducers as shown in Fig. 6. Moreover, our results provide novel insights into the communication between target cells and AdMSC-derived exosomes. Therapy using exosomes could be a safer and more efficient cell-free therapy for promoting anti-inflammation and angiogenesis, which will provide a theoretical and experimental basis for new stem cell-based therapies to treat immune and vasculogenesis-related diseases. Recently, several studies have shown that physical or chemical pretreatment can increase exosomal effects^31,39^. Future studies should determine if we can enhance the function of exosomes with small molecule compounds, and if the improved exosomes efficiently function in animal models.

## Methods

### Cells and cell culture

AdMSCs and HUVECs were purchased from CEFO (Seoul, Republic of Korea). AdMSCs were cultured in Dulbecco’s Modified Eagle Medium (DMEM)-low glucose (Invitrogen, Carlsbad, CA, USA) supplemented with 10% fetal bovine serum (FBS, Invitrogen) and 1% penicillin/streptomycin (P/S, Invitrogen) at 37 °C in a 5% CO_2_ environment. HUVECs were cultured in EGM-2 endothelial cell growth Medium-2 (Lonza, Walkersville, MD, USA) supplemented with 10% FBS and 1% P/S at 37 °C in 5% CO_2_. The culture medium was exchanged every 3 or 4 days until the cells reached approximately 90% confluence. Before reaching 100% confluence, the cells were sub-cultured at a 1:3 or 1:4 ratios for cell expansion and further experiments. To obtain exosomes derived from AdMSCs, AdMSCs were cultured in DMEM containing 5% exosome-depleted FBS (Invitrogen). In this study, AdMSCs from 3 donors (33-, 26- and 29-year-old females) were used.

### Differentiation assay

To assess the differentiation capacity of AdMSCs, the cultured cells were induced in osteogenic, adipogenic, and chondrogenic differentiation medium (Lonza) for 3 weeks. The medium was changed every 3 or 4 days, and 10 ng/mL of TGF-β3 (Lonza) was added to the cells for chondrogenesis. To confirm differentiation capacity, the differentiated cells were stained with von Kossa for osteogenesis, Oil Red O for adipogenesis, and Safranin-O for chondrogenesis. Images of the stained cells were obtained using an inverted phase microscope (Olympus-IX71, Olympus, Tokyo, Japan).

### Flow cytometry analysis

For immunophenotyping the AdMSCs, the cells were harvested using 0.05% trypsin/EDTA (Invitrogen) at a single cell level and incubated on ice for 30 min with the following monoclonal antibodies: anti-CD29, anti-CD34, anti-CD44, anti-CD45, anti-CD73, and anti-CD90 (BD Pharmingen, San Diego, CA, USA). After washing with phosphate-buffered saline (PBS, Invitrogen), the cells were analyzed using a Cytomics Flow Cytometer (Beckman Coulter, Fullerton, CA, USA).

### Isolation and characterization of exosomes from AdMSCs

Exosomes were isolated from the AdMSC supernatant using an exosome isolation kit (System Biosciences, Palo Alto, CA, USA) according to the manufacturer’s instructions. Briefly, the culture medium was harvested and centrifuged at 1500 × g for 5 min to remove cell debris. Then, the supernatant was transferred to a fresh conical tube, and the ExoQuick-TC reagent was mixed with the supernatant (1:5 ratio). After inverting four times, the mixture was incubated overnight at 5 °C. The next day, the mixture was centrifuged at 1500×*g* for 30 min, and the supernatant was removed. The isolated exosomes were resuspended in PBS (Invitrogen). The exosomes were quantified using a BCA protein assay kit (Invitrogen), followed by stored at -80 ℃. Transmission electron microscopy (TEM, JEM-1011, JEOL, Tokyo, Japan) was used to observe the morphology of the exosomes. To examine the size distribution, the exosomes were evaluated using a nanoparticle tracking system according to the manufacturer’s protocols (Nanosight NS300, Malvern Panalytical, Malvern, UK).

### Western blot

Total proteins were extracted using RIPA lysis buffer (Biosesang, Seongnam, Republic of Korea) containing a protease inhibitor cocktail (Sigma Chemical Co., St. Louis, MO, USA). Protein concentration was quantified using a BCA protein assay kit (Invitrogen). Briefly, proteins were fractionated on a 12% sodium dodecyl sulfate polyacrylamide gel and transferred onto a polyvinylidene fluoride membrane (Bio-Rad Laboratories, Redmond, WA, USA). After blocking with 5% skim milk (BD-Pharmingen) for 1 h, samples were incubated with primary antibodies against the following proteins: anti-CD9 (1:500, LSBio #C119436, Seattle, WA, USA), anti-CD63 (1:500, LSBio #B16793), anti-CD81 (1:1000, System Biosciences #EXOAB-KIT-1), anti-HSP70 (1:1000, System Biosciences #EXOAB-KIT-1), anti-ROCK1 (1:1000, Cell Signaling Technology #4035, Danvers, MA, USA), anti-PTEN (1:1000, Abcam #ab137337, Cambridge, UK), and anti-β-actin (1:1000, Santa Cruz Biotechnology #sc47778, Inc.) for 24 h at 4 °C. Subsequently, the samples were incubated with a horseradish peroxidase-conjugated anti-mouse secondary antibody (1:1000, GeneTex #GTX221667-01, CA, USA) and anti-rabbit secondary antibody (1:1000, GeneTex #GTX221666-01) for 1 h at room temperature. After washing three times for 5 min, the membranes were developed using SuperSignal West Femto Maximum Sensitivity Substrate (Invitrogen) according to the manufacturer’s instructions. Signals were detected using the LAS4000 system (GE Healthcare, Uppsala, Sweden).

### Real-time quantitative polymerase chain reaction

Total RNA was extracted using the RiboEx reagent (GeneAll, Seoul, Republic of Korea). After analyzing the quality and quantity of RNA using a NanoDrop ND-1000 spectrophotometer (Thermo Fisher Scientific, Waltham, MA, USA), 500 ng of RNA was used for reverse transcription using HiSenScript™ RH[-] cDNA synthesis kit (iNtRON, Seongnam, Republic of Korea), according to the manufacturer’s instructions. Real-time qPCR was performed using a Light Cycler 480 II SYBR Green I Master mix (Roche Molecular Systems, Pleasanton, CA, USA) under specific conditions. Experiments were performed in triplicates using the primers listed in Table S1. Relative mRNA expression levels were calculated using the comparative C_T_ method, and the values were normalized to glyceraldehyde-6-phosphate dehydrogenase (*GAPDH*). For miRNA analysis, the U6 primer was used as an internal control.

### miRNA inhibitor transfection and inhibition assays

To test the inhibitory effects of ROCK1 and PTEN, 5 × 10^5^ THP-1 cells were plated in 6-well plates (Nunc, Roskilde, Denmark) and cultured with 10 nM ROCK inhibitor (Sigma Chemical Co., Y-27632) and 1 nM PTEN inhibitor (Sigma Chemical Co., SF1670) for 24 h. After washing with PBS, the cells were harvested for real-time qPCR analysis. Lipofectamine 2000 (Invitrogen) was used to transfect miR-132 and miR-146a inhibitor (Sigma Chemical Co.) into HUVECs, according to the manufacturer’s protocol. Briefly, 2.5 × 10^5^ HUVECs were seeded on a 6-well plate (Nunc) and cultured with 50 nM inhibitor for 24 h. Transfected cells were subjected to real-time qPCR and angiogenesis assay using Matrigel.

### Cell proliferation assays

To examine the effects of exosomes on HUVEC proliferation, 1 × 10^3^ cells were seeded with 5 μg/mL of exosomes in a 96-well plate (BD Falcon, Swedesboro, NJ, USA). After 24 h, the proliferation rate was evaluated using a WST-based assay kit (EZ-Cytox, Daeil Lab, Seoul, Republic of Korea), according to the manufacturer’s instructions. The absorbance of proliferation activity was measured at 450 nm using a microplate reader (Molecular Devices, San Jose, CA, USA). Untreated cells were used as controls, and the absorbance of treated cells was normalized to that of control cells.

### Tube formation assay

Capillary network formation was evaluated using a tube formation assay in Matrigel (BD Pharmingen). 2 × 10^5^ of HUVECs were seeded onto Matrigel-coated 24-well plates (BD Falcon) and cultured in EGM-2 supplemented with 10% FBS and 1% P/S for 24 h at 37 °C in 5% CO_2_. *The *in vitro tube formation was analyzed by microscopy (Olympus).

### Statistical analysis

Data are expressed as the mean ± standard deviation (SD). One-way ANOVA with Tukey’s test and t-test were performed for comparisons (Prism ver. 7, Graph Pad Software, Inc., La Jolla, CA). Statistical significance was defined as *p* < 0.05, *p < 0.05, and **p < 0.01.

## Supplementary Information

Supplementary Information.

## Data Availability

All the data analyzed in this study are included in this published article. The data used to support the findings of this study are available from the corresponding author upon request.
