# Supplementary material for: Human adipose mesenchymal stem cells modulate inflammation and angiogenesis through exosomes
Source: Sci Rep. 2022 Feb 17;12:2776. doi: 10.1038/s41598-022-06824-1 (PMC8854709; doi:10.1038/s41598-022-06824-1)
Supplement: Supplementary file 1 — Supplementary Information. [file 41598_2022_6824_MOESM1_ESM.docx]

Supplementary information

**Human adipose mesenchymal stem cells modulate inflammation and angiogenesis through exosomes**

*June Seok Heo^1^, Sinyoung Kim^1,2,*^*

*^1^* Dr. J. S. Heo

Cell Therapy Center, Severance Hospital, 50-1 Yonsei-ro, Seodaemun-gu, Seoul 03722, South Korea

E-mail: juneseok@yuhs.ac

*^1,2^* Prof. S. Kim

Cell Therapy Center, Severance Hospital, Yonsei University College of Medicine, Department of Laboratory Medicine, Yonsei University, 50-1 Yonsei-ro, Seodaemun-gu, Seoul 03722, South Korea E-mail: [SYKIM@yuhs.ac](mailto:SYKIM@yuhs.ac)


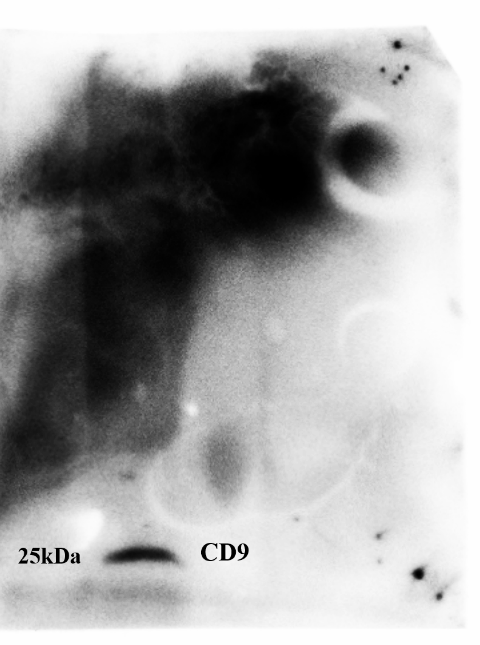


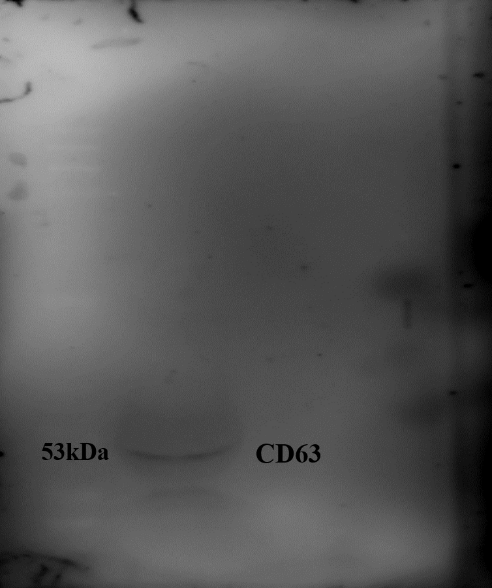


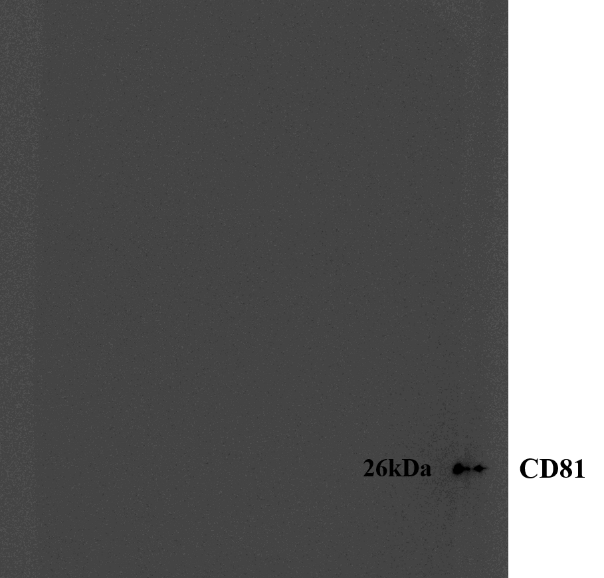


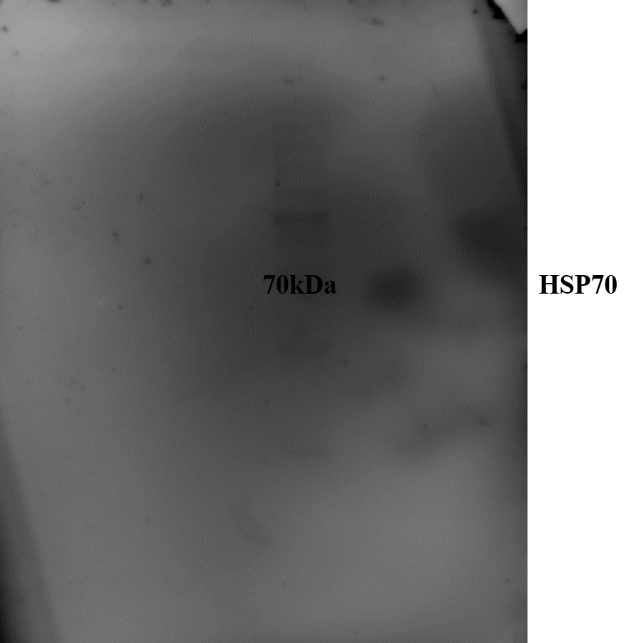


**Supplementary figure 1.** CD9, CD63, CD81 and HSP70 of exosomal markers were detected in AdMSCs-derived exosomes by Western blot assay.
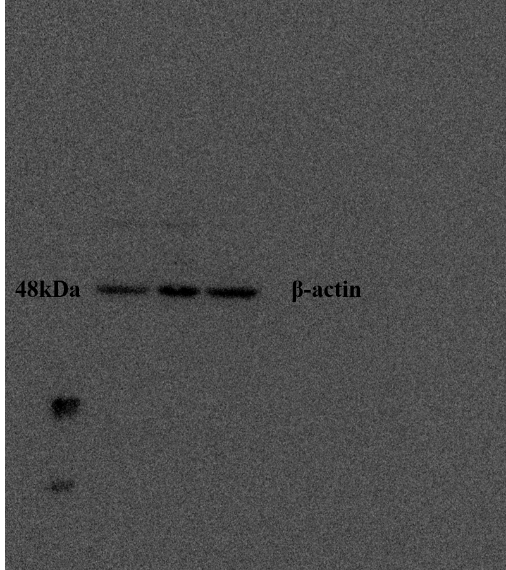


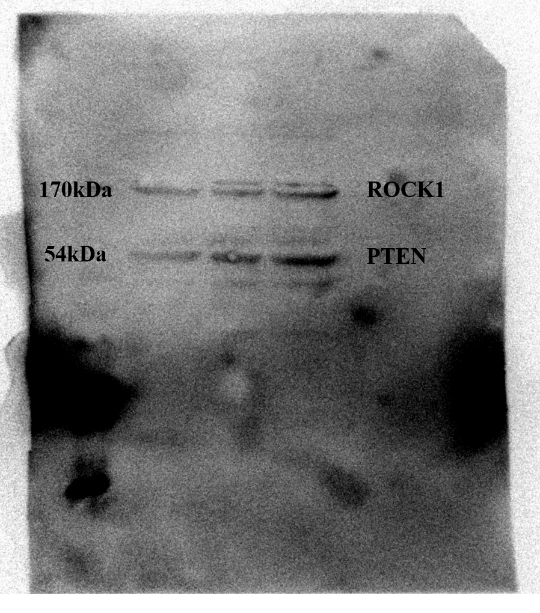


**Supplementary figure 2.** The protein levels of ROCK1 and PTEN were confirmed by Western blotting. β-actin was used as an internal control.


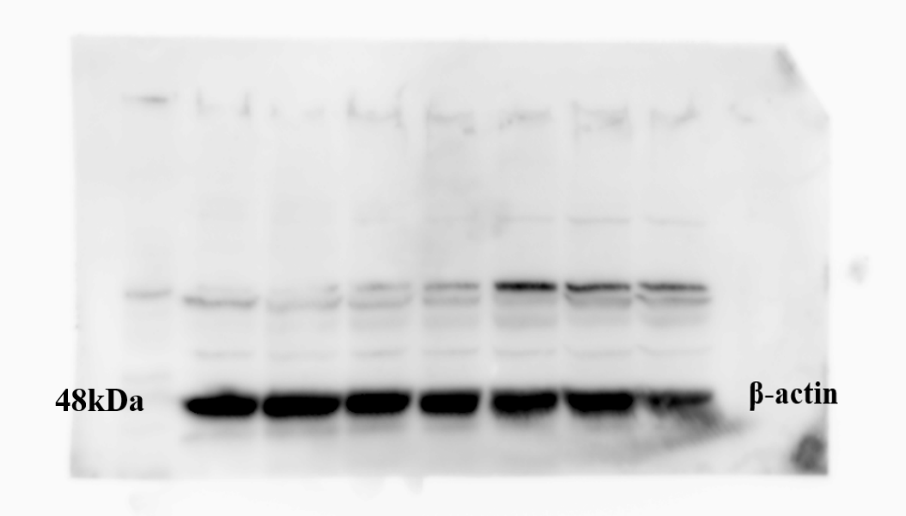


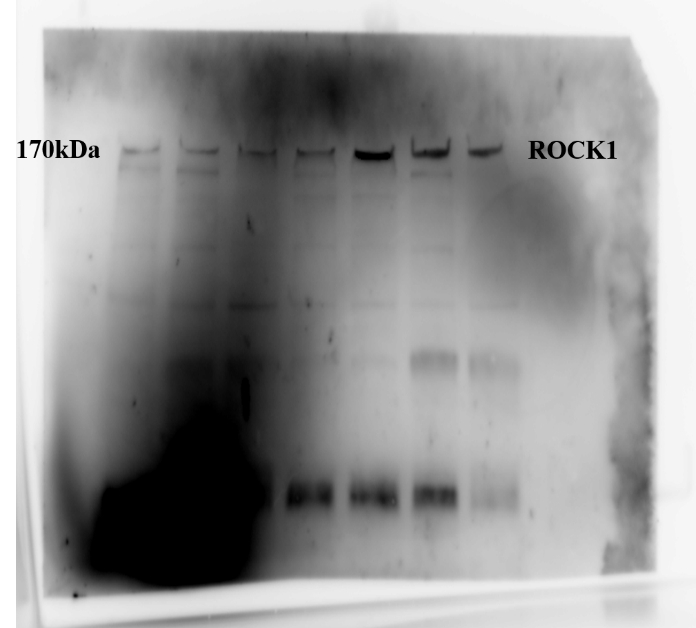


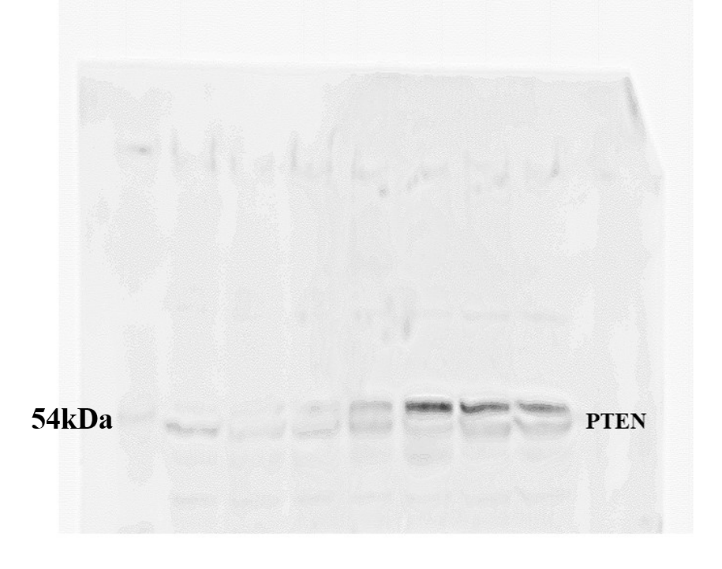


**Supplementary figure 3.** Protein levels of ROCK1 and PTEN were analyzed by western blot. β-actin was used as controls.

**Supplementary table 1.** Primers sequences.

| **Primer name** | **Sequence** |
| --- | --- |
| *GAPDH* | F: ACCCACTCCTCCACCTTTGA  R: CTGTTGCTGTAGCCAAATTCGT |
| U6 | F: CTCGCTTCGGCAGCACATATACT  R: ACGCTTCACGAATTTGCGTGTC |
| *TNF-α* | F: TTGAGGGTTTGCTACAACATGGG  R: GCTGCACTTTGGAGTGATCG |
| *IL-6* | F: AGACAGCCACTCACCTCTTCAG  R: TTCTGCCAGTGCCTCTTTGCTG |
| *IL-8* | F: AAGAGAGCTCTGTCTGGACC  R: GATATTCTCTTGGCCCTTGG |
| *CD163* | F: CGGCTGCCTCCACCTCTAAGT  R: ATGAAGATGCTGGCGTGACA |
| *ARG1* | F: ACAGTTTGGCAATTGGAAGCA  R: CACCCAGATGACTCCAAGATCAG |
| *CD206*  (Mannose receptor) | F: TTCGGACACCCATCGGAATTT  R: CACAAGCGCTGCGTGGAT |
| *TGF-β1* | F: TACCTGAACCCGTGTTGCTCTC  R: GTTGCTGAGGTATCGCCAGGAA |
| *IL-10* | F: TCTCCGAGATGCCTTCAGCAGA  R: TCAGACAAGGCTTGGCAACCCA |
| *ROCK1* | F: ACCTGTAACCCAAGGAGATGTG  R: CACAATTGGCAGGAAAGTGG |
| *PTEN* | F: CGGCAGCATCAAATGTTTCAG  R: AACTGGCAGGTAGAAGGCAACTC |
| *ANGIOPOIETIN1(ANGPT1)* | F: CTTCAAGGCTTGGTTACTCGTC  R: CTCTTCCTCTCTTTTTCCTCCC |
| *FLK1(KDR)* | F: AACGACTGCCTTATGATGCC  R: ACTGTCCTGCAAGTTGCTGTC |
| *VASOHIBIN-1(VASH1)* | F: CTCAAGATTGGCAAAGGGACG  R: TTTCACTGCGGCTGTTCCTGC |
| *THROMBOSPONDIN-1(THBS1)* | F: GGTGATGTATGAAGGGAAGAA  R: GGAAGCCAAGGAGAAGTGAT |
| miR-124-3p | F: TAAGGCACGCGGTGAATGCC  R: GATTGAATCGAGCACCAGTTAC |
| miR-132 | F: CCAGCATAACAGTCTACAGCCA  R: TATGGTTGTTCACGACTCCTTCAC |
| miR-146a | F: TAACCGAATCTTGCCATACGCA  R: CGGAGTCTGAGAACTGAATTCCA |
